# LUBAC and ABIN-1 Modulate TRAIL-Based NF-κB Induction in Human Embryonic Kidney 293 Cells

**DOI:** 10.1089/biores.2018.0006

**Published:** 2018-05-01

**Authors:** Sebastian Dorn, Christian Schoergenhofer, Michael Krainer, Markus Müller, Bernd Jilma

**Affiliations:** ^1^ Department of Clinical Pharmacology, Medical University of Vienna, Vienna, Austria.; ^2^ Clinical Division of Oncology, Department of Internal Medicine I, Medical University of Vienna, Vienna, Austria.

**Keywords:** ABIN-1, HEK 293 cells, NF-κB regulation, LUBAC, TRAIL

## Abstract

Tumor necrosis factor (TNF)-related apoptosis-inducing ligand (TRAIL) is known to activate the canonical NF-κB pathway similar to TNF. The exact mechanism of the entire signaling cascade is still under investigation. The involvement of linear ubiquitylation as upregulating component has already been shown recently in some cell lines, but not in human embryonic kidney 293 (HEK293) cells. The downregulating function of the ABIN-1 (A20 binding and inhibitor of NF-κB) as linear ubiquitylation antagonist has been shown in combination with some NF-κB-inducing pathways, but not with TRAIL. We performed luciferase and western blot assays using HEK293 cells stimulated with either TRAIL (or TNF as a control) to analyze the involvement of linear ubiquitin chain assembly complex (LUBAC) components and the impact of ABIN-1 and ABIN-1-MAD (truncated form without A20 binding site) on NF-κB signaling. For overexpression experiments, we added plasmids of ABIN-1 and ABIN-1-MAD or LUBAC components HOIP, HOIL-1, or SHARPIN (single and combinations). For downregulation experiments five pairs of either SHARPIN, HOIL-1, or HOIP targeting miRNAs or one miRNA for ABIN-1 were designed and added. ABIN-1 and its truncated form ABIN-1-MAD reduced the NF-κB induction significantly indicating its involvement as antagonist (independent of deubiquitinase A20) of linear ubiquitylation in TRAIL-induced NF-κB signaling. In opposition, knockdown of ABIN-1 using a specific ABIN-1 miRNA led a clear increase of NF-κB signaling. Addition of single LUBAC components or combinations (except for SHARPIN with HOIL-1) resulted in clearly stronger NF-κB inductions. MiRNAs targeting LUBAC components significantly reduced NF-κB activation. Thus, in HEK293 cells linear ubiquitylation by LUBAC critically upregulates and ABIN-1 downregulates TRAIL-induced NF-κB signaling and may be interesting targets for future pathological therapies.

## Introduction

Various regulator pathways and proteins exist that may influence apoptotic pathways that is, NF-κB, MAP-kinases or inhibitor of apoptosis proteins (IAP proteins).^1,2^ The canonical NF-κB pathway modulates the expression of apoptotic regulators which may support or inhibit cellular apoptosis.^3^ This pathway is activated by TNF, which leads to the assembly of the TNF receptor 1 (TNFR1)-associated signaling complex, containing TNFR-1, TRAF2, RIP1, cIAP 1/2, and TRADD and the recruitment of IκB kinases IKKα, IKKβ, and IKKγ (NEMO).^1,4–6^ The recruitment of these kinases is supported by the formation of Lys11- and Lys63-based polyubiquitination chains, which serve as stable platforms.^7^

The E3 ligase linear ubiquitin chain assembly complex (LUBAC) consisting of the components SHARPIN, HOIL-1, and HOIP interacts with IKKγ and ensures persistent NF-κB activation.^8–11^ Among other functions, NF-κB upregulates the antiapoptotic molecules c-FLIP(s), which directly inhibit the cleavage of the procaspase-8 into its active form, and c-FLIP(L), which interferes with the release and maturation of caspase-8 from the DISC.^12–16^

The TNF-related apoptosis-inducing ligand (TRAIL) also activates the NF-κB pathway in a similar way as TNF. When TRAIL binds to its receptors TRAIL-receptor TRAIL-R1 or TRAIL-R2, the DISC consisting of FADD, RIP1, c-FLIP, cIAP1 and 2, Procaspase 8, and probably TRAF2 is rapidly assembled.^17,18^ Subsequently RIP1 is ubiquitylated by cIAP1/2. It accumulates within the DISC and is directly involved in the stimulation of NF-κB signaling in combination with caspase-8.^7,19,20^ This pathway leads to the phosphorylation of IKKβ (and potentially IKKα).

These similarities and the fact that in many tumor cells TRAIL-R1 and TRAIL-R2 are expressed at the cell surface led to the hypothesis that LUBAC could also be involved in TRAIL-based NF-κB stimulation and could result in a stronger NF-κB induction.^21^ In combination with TRAIL-induced NF-κB signaling, an antagonistic role of deubiquitinase A20 by destabilizing branched as well as linear polyubiquitylation chains associated with critical signaling components as RIP1 and NEMO and promoting RIP1 degradation performing ligase activity is under discussion.^22^ These potential functions make it to a key factor in NF-κB signaling inhibition.

In comparison in TNF signaling ABIN-1 can potentially act as efficient NF-κB signaling inhibitor either indirectly as adaptor for A20^23,24^ or directly by binding to polyubiquitylated chains preventing binding of RIP1, NEMO, or TRAF6 and NF-κB upregulation.^25^ These features and its ability to bind highly specific to linear ubiquitylation chains may make it to an attractive player in TRAIL-induced NF-κB signaling.

Since an involvement of LUBAC and of ABIN-1 and ABIN-1-MAD in TNF-induced NF-κB signaling has been shown in human embryonic kidney 293 (HEK293) cells^25–28^ and also the involvement of LUBAC in TRAIL-based NF-κB signaling in mouse embryonic fibroblasts, K562, HeLa, HCT116, MCF7, HT29, and A549 cells,^29^ we did our experiments in HEK293 cells. HEK293 cells are a very well-characterized cell line with features of kidney epithelial and adrenal gland cells, which has often been used as model for studying cancer-associated genes.^30^ Its immortalized status and ability to form tumors in mice after injection^31^ show its tumorigenic character and makes it to a good tumor *in vitro* cell model reflecting some important characteristics of tumor cells.

## Methods

### Luciferase assay

0.3 × 10^4^ HEK293 cells were seeded in poly-D-lysine (P6405-5 MG; Sigma) coated 96-well plates and incubated overnight. On the next day, the cells were transfected by using 0.1 μL Turbofect (R0531; Fermentas) in 40 μL serum-free medium and the reaction stopped after 2 h. For overexpression experiments 75 ng NF-κB responsive reporter (#210978; Stratagene), 10 ng pRL-CMV vector (E2261; Promega) in combination with 10, 20, or 30 ng pCAGGS-E-hABIN-1 (LMBP 5126) or 10, 20, 30 ng pCAGGS-E-hABIN-1-MAD (LMBP 5131) from BCCM or 10 ng HOIP-Myc, 10 ng HA-HOIL-1, or 10 ng FLAG-SHARPIN (gifts from K. Iwai) were cotransfected. As control, the plasmid pBluescript II KS (+) (#212207; Stratagene) was used.

For downregulations, five pairs of oligonucleotides for SHARPIN, HOIL-1, and HOIP and one for ABIN-1 were designed (Life technologies; Supplementary Data) and cloned into POL II MIR RNAI GFP vectors (K493600; Life Technologies). For experiments in addition to 25 ng of the reporter and 10 ng of the Renilla luciferase expressing constructs, 100 ng of the miRNA expression vectors were cotransfected and the GFP expressions were analyzed. As controls, the miRNA negative control vector (from K493600) and the pBluescript II KS (+) plasmid were used.

In all experiments, 48 h after, transfection media with 100 ng/mL TNF-α (T-6674-10UG; Sigma) or 1000 ng/mL TRAIL (616374; Merck Chemicals) were added to the cells, and 24 h later, they were lysed and the expression levels of firefly luciferase and Renilla luciferase were analyzed by using the dual luciferase reporter assay system and the instructions from Promega (E1960) and the Victor™ Multilabel Plate Reader (Perkin Elmer).

### Western blot

0.125 × 10^5^ HEK293 cells were seeded on Poly-D-Lysine-coated six-well plates and transfected with 3 μg Bluescript II KS vector, 3 μg negative miRNA control plasmid as control or 3 μg of the different SHARPIN, HOIP, HOIL-1, or ABIN-1 miRNA plasmids or 300 ng of HOIP-Myc, HA-HOIL-1, FLAG-SHARPIN or 300 ng, 600 ng or 900 ng of pCAGGS-E-hABIN-1 or 300 ng, 600 ng or 900 ng of pCAGGS-E-hABIN-1-MAD by using 3 μL Turbofect in 1.2 mL serum-free medium. Again to the cells 100 ng/mL TNF-α (for 30 min) or 1000 ng/mL TRAIL (for 50 min) was added after 48 h. Subsequently, the cells were lysed by using a lysis buffer, containing PhosSTOP (0490683701; Roche) and protease inhibitor (04693116001; Roche), and the protein amounts were measured by Bradford assay, the proteins denatured and stored at −80°C.

The different protein expression levels were analyzed by using 5% stacking and 13% separating SDS-PAGE gels, PDVF membranes (W5601; Millipore), horseradish peroxidase detection reagents (32106; Thermo Scientific), and Hyperfilms (28-9068-40; Amersham). The stripping process was done in combination with restore Plus western blot stripping buffer (46430; Thermo Scientific). For the detection of the protein expressions, primary antibodies for SHARPIN (ab69507; Abcam), HOIP (ab85294; Abcam), HOIL-1 (ab38540; Abcam), ABIN-1 (ab70152; Abcam), IκB-α (sc-203; Santa Cruz), Phospho-IκBα (Ser32/36; #9246; Cell Signaling), α-Tubulin (#2125; Cell Signaling), and β-Actin (#8457; Cell Signaling) were used.

The intensity of the NF-κB bands were normalized to the corresponding Actin or Tubulin signals, then the normalized signals were used to calculate fold induction, while with the negative control, vector-transfected samples were arbitrary set as 1. For the band quantifications, the program Image Studio Lite version 5.2 was used.

## Results

First, we examine the potential influence of ABIN-1 on TRAIL-induced NF-κB signaling. For this, HEK293 cells were transfected with a plasmid overexpressing ABIN-1 and stimulated with 1000 ng/mL TRAIL (TRAIL concentration was chosen according to observed stable NF-κB induction in experiments being relevant for establishing possible resistance in HEK 293cells.^32–35^

**FIG. 1.**
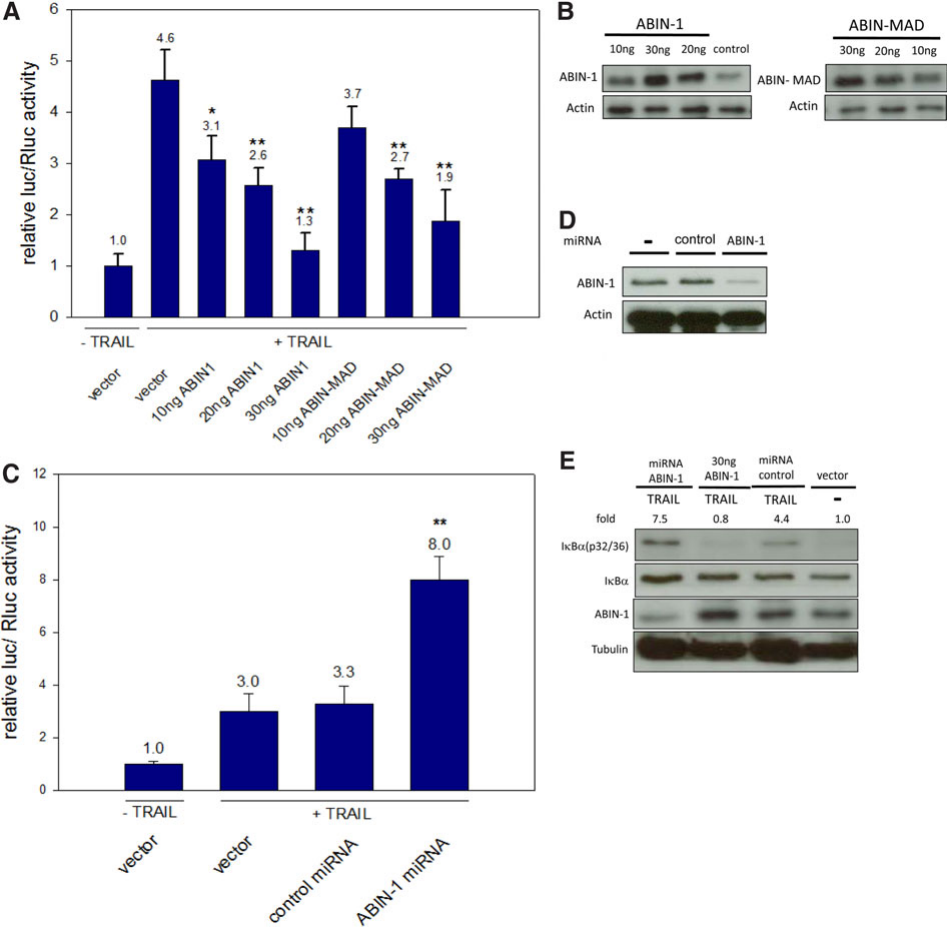
ABIN-1 and ABIN-MAD inhibit NF-κB signaling upon TRAIL treatment. ABIN-1, ABIN-MAD plasmids **(A)**, ABIN-1-targeting miRNA **(C)**, or Bluescript vector as control **(A, C)** in combination with a firefly luciferase expressing NF-κB responsive reporter, and a Renilla luciferase expressing vector were cotransfected in HEK293 cells. After 48 h, 1000 ng/mL TRAIL was added, and the cells were lysed 24 h later to measure the relative luciferase levels. All values from relative firefly/Renilla luciferase assay represent three independent experiments with four **(A)** or three **(C)** replicants (per each construct and the bars indicate standard deviations. The statistical calculations comparing the transcriptional NF-κB inductions of Bluescript vector with different plasmid amounts of ABIN-1, ABIN-MAD, or ABIN-1 miRNA were done by Student's *t*-test (one asterisk indicates *p* ≤ 0.05, two asterisks *p* ≤ 0.01). HEK293 cells were transfected with Bluescript vector **(B, D, E)**, ABIN-1 **(B, E)**, ABIN-MAD **(B)**, ABIN miRNA **(D, E)**, or control miRNA **(D, E)** and treated with TRAIL (1000 ng/mL for 50 min) or left untreated. The expression levels of ABIN-1 **(B, D, E)**, ABIN-MAD **(B)**, phosphorylated IκBα (32/36), unphosphorylated IκBα **(E)** β-Actin **(B, D)**, and α-Tubulin proteins **(E)** were evaluated by western blotting. The expression level of α-Tubulin **(E)** was evaluated after membrane stripping. Numbers indicate ratios of signal intensities of phosphorylated IκBα (32/36) to unphosphorylated IκBα, normalized to the ratio of untreated cells (arbitrarily set to 1.0). For determination of intensities, the software Image Studio Lite was used. ABIN-1, A20 binding and inhibitor of NF-kappaB; ABIN-MAD, minimal active domain of ABIN; TRAIL, TNF-related apoptosis-inducing ligand.

Using a dual luciferase assay with a NF-κB responsive reporter increasing amounts, ABIN-1 resulted in decreasing transcriptional NF-κB activity (Fig. 1A). To test whether ABIN-1 can potentially downregulate TRAIL-induced NF-κB signaling without A20 interaction, a truncated form of ABIN-1, ABIN-MAD (aa 441–601, was overexpressed, which is not able to interact with A20 anymore. Again a reduced NF-κB signaling activity was observed with increasing amounts of plasmids of ABIN-MAD (Fig. 1A). In a subsequent experiment by using an ABIN-1-specific miRNA knockdown of ABIN-1 (for miRNA sequence see Supplementary Data) in combination with TRAIL, a clear increase of transcriptional NF-κB activity was observed (Fig. 1C).

Subsequently to these experiments IκBα phosphorylation was analyzed by western blotting as a clear indicator of canonical NF-κB activation. From literature, it is known that both IκBα residuals 32 and 36 are phosphorylated in case of TNF addition being critical for IκBα degradation and canonical NF-κB induction.^27^ The phosphorylation of the same sites of IκBα is also important for the TRAIL-based NF-κB activation. According to luciferase experiments before, downregulation of ABIN-1 resulted in an increase and overexpression of ABIN-1 in a clear reduction of IκBα phosphorylation.

These results clearly indicate that ABIN-1 has the ability to inhibit TRAIL-based NF-κB signaling also without the deubiquitinase A20. Thus, since it is known that ABIN-1 binds strongly and specifically to linear ubiquitylation chains, an involvement of LUBAC in TRAIL-stimulated NF-κB signaling in HEK293 cells is likely. Lafont and colleagues showed that HOIP is critically involved in TRAIL-based NF-κB induction, but the roles of SHARPIN and HOIL-1 were not studied in detail.^29^ To test the hypothesis of LUBAC involvement and to investigate which of the LUBAC members, HOIP, HOIL-1, or SHARPIN, are most essential for NF-κB induction, we conducted further experiments.

**FIG. 2.**
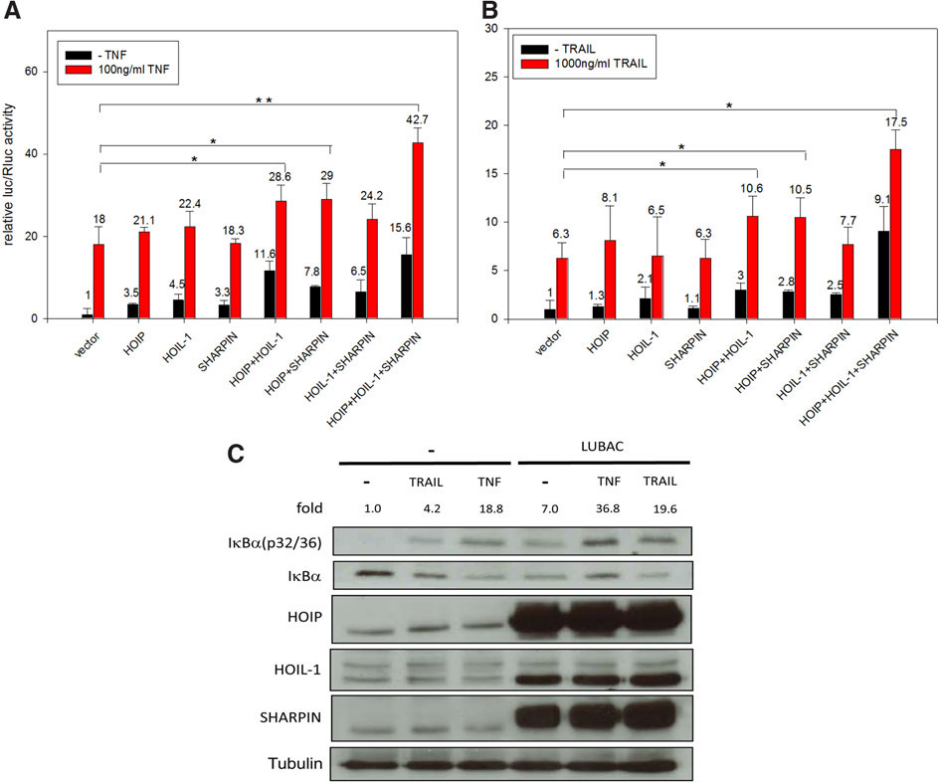
LUBAC further increases NF-κB signaling upon TRAIL treatment. HEK293 cells were cotransfected with Bluescript vector or different combinations of HOIP-Myc, HA-HOIL-1, or SHARPIN expressing plasmids in combination with firefly luciferase expressing NF-κB responsive reporter and Renilla luciferase expressing construct and treated after 48 h with **(A)** TNF (100 ng/mL) or **(B)** TRAIL (1000 ng/mL) for further 24 h or left untreated to analyze the resulting relative luciferase activity. **(C)** HEK293 cells were transfected with Bluescript vector as a control or with LUBAC (HOIP-Myc, HA-HOIL-1, and FLAG-SHARPIN-containing plasmids) and treated with TNF (100 ng/mL, 30 min) or TRAIL (1000 ng/mL, 50 min) or left untreated. The results of the relative firefly/Renilla luciferase assay are derived from three independent experiments with three replicants per each construct and the bars characterize standard deviations. One asterisk represents a significance level of *p* ≤ 0.05 and two asterisks of *p* ≤ 0.01 (The differences in NF-κB inductions between vector and LUBAC components with TNF or TRAIL were determined by Student's *t*-test). The expression levels of LUBAC components and phosphorylated IκBα (32/36) and unphosphorylated IκBα proteins were evaluated by western blotting. The expression levels of α-Tubulin and SHARPIN were analyzed after membrane stripping. Numbers indicate ratios of signal intensities of phosphorylated IκBα (32/36) to unphosphorylated IκBα, normalized to the ratio of untreated cells without overexpressed LUBAC components (arbitrarily set to 1.0). The determination of intensities was done by using the software Image Studio Lite. LUBAC, linear ubiquitin chain assembly complex.

In a first step as control, the several LUBAC members HOIP, HOIL-1, and SHARPIN were overexpressed without and with 100 ng/mL TNF (TNF concentration is a common amount to investigate an effect of TNF signaling components on TNF induction in HEK293 cells^33,36,37^ to confirm known results that LUBAC is able to potentiate NF-κB signaling upon TNF application (Fig. 2A). Similar to former publications, the application of each component alone led to no or weak NF-κB induction, but the combination of two or all three members (except SHARPIN with HOIL-1) resulted in a much stronger upregulation even without addition of TNF.^9^ In addition, the application of TNF in combination with the several LUBAC components showed further NF-κB upregulation demonstrating that LUBAC is a key player in TNF-based NF-κB activation.

The same experiments were repeated in combination with TRAIL to analyze whether and which LUBAC components are involved in TRAIL-based NF-κB induction (Fig. 2B). Overexpression of the LUBAC members alone led to no or weak NF-κB upregulation, but combinations resulted in clear activation (except HOIL-1 with SHARPIN) and the highest one with all three proteins. By adding TRAIL and the several LUBAC components again around six to eightfold inductions could be shown in comparison to the overexpressed components without TRAIL. The combined overexpression of the LUBAC proteins together with TRAIL (except HOIL-1 and SHARPIN) significantly increased NF-κB signaling in comparison to overexpressed LUBAC components alone.

Analyzing the phosphorylation state of IκBα residuals, 32 and 36 upon TNF treatment as control, a strong phosphorylation, and in case of TRAIL, a weak but clear phosphorylation of IκBα was visible (Fig. 2C). The overexpression of LUBAC components resulted in a stronger IκBα phosphorylation than in the experiments without LUBAC, even without TRAIL or TNF stimulation. Finally, the combination of TNF or TRAIL with LUBAC overexpression followed a clear increase of IκBα phosphorylation.

Thus, we were able to show that LUBAC contributes to an upregulation of NF-κB signaling upon TRAIL treatment. As a next step, we wanted to analyze whether the several LUBAC components do not just contribute but are required for this process. For demonstrating that again in combination with dual luciferase assay (including a NF-κB responsive reporter) and TNF/TRAIL treatment, five different SHARPIN, HOIL-1, and HOIP-targeting miRNAs were expressed, which should result in clear downregulations of NF-κB signaling (for miRNAs sequences see Supplementary Data).

**FIG. 3.**
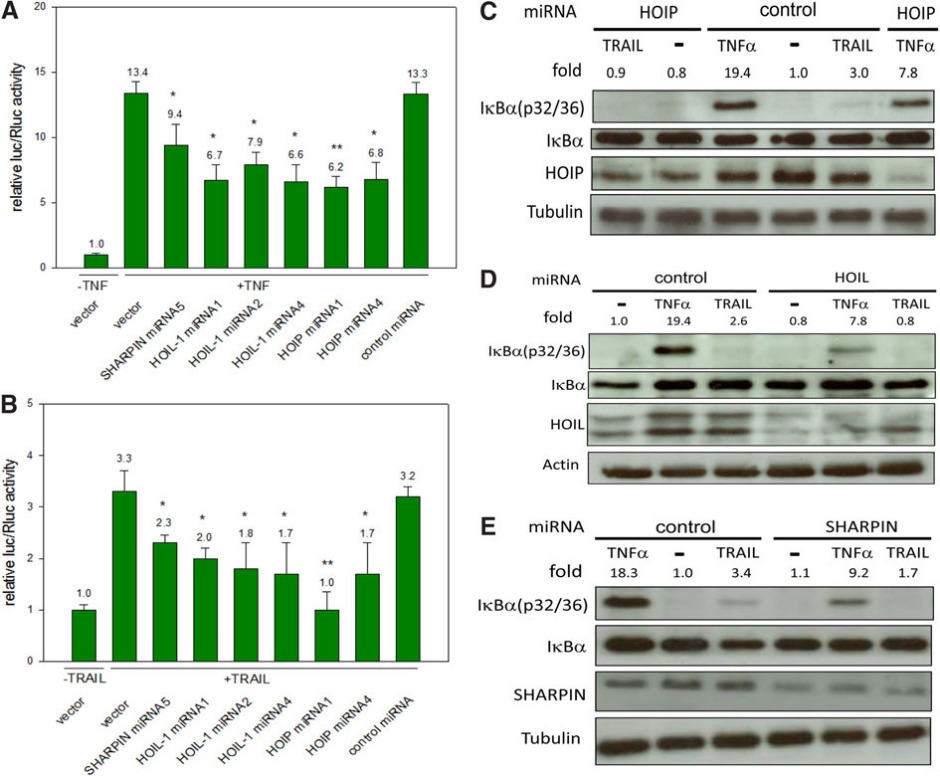
Downregulations of HOIP, HOIL-1, or SHARPIN decrease TRAIL-based NF-κB signaling. Various HOIP, HOIL-1, or SHARPIN-targeting miRNAs, a control miRNA, or Bluescript vector were cotransfected with the firefly luciferase expressing NF-κB responsive reporter and the Renilla luciferase expressing vector to evaluate the resulting relative luciferase expressing levels after 24 h in untreated or **(A)** TNF- (100 ng/mL, 48 h after transfection) or **(B)** TRAIL (1000 ng/mL, 48 h after transfection)-treated HEK293 cells. The results of the relative firefly/Renilla luciferase assay are derived from three independent experiments with three replicants per each construct and the bars characterize standard deviations. One asterisk represents a significant level of *p* ≤ 0.05 and two asterisks of *p* ≤ 0.01 (The differences in NF-κB inductions between vector and LUBAC components with TNF or TRAIL were determined by Student's *t*-test). HEK293 cells were transfected with Bluescript vector, **(C)** HOIP miRNA1, **(D)** HOIL-1 miRNA1, or **(E)** SHARPIN miRNA and treated with TNF (100 ng/mL for 30 min) or TRAIL (1000 ng/mL for 50 min) or left untreated. The expression levels of HOIP, HOIL-1, SHARPIN, phosphorylated IκBα (32/36), unphosphorylated IκBα, and α-Tubulin were analyzed by western blotting. The expression levels of β-Actin **(D)** and SHARPIN **(E)** were evaluated after membrane stripping. Numbers indicate ratios of signal intensities of phosphorylated IκBα (32/36) to unphosphorylated IκBα, normalized to the ratio of untreated cells with control miRNA (arbitrarily set to 1.0). For determination of intensities, the software Image Studio Lite was used.

In first experiments, HEK293 cells were stimulated by TNF as control and the impact of differently expressed miRNAs for SHARPIN, HOIL-1, and HOIP knockdowns on NF-κB signaling were analyzed. Actually, by using miRNAs against SHARPIN (miRNA5), HOIL-1 (miRNA1, 2, 4), and HOIP (miRNA1, 4), a clear reduction of TNF-based NF-κB signaling was observed (Fig. 3A), confirming that LUBAC is essential for the NF-κB activation upon TNF treatment. The outstanding miRNAs did not show any NF-κB reduction (data not shown).

These experiments were then conducted using TRAIL as a stimulus with similar results (Fig 3B). Again the same miRNAs led to reductions in NF-κB signaling.

To demonstrate the impact of downregulation of SHARPIN, HOIL-1, or HOIP on NF-κB signaling on protein level, the resulting phosphorylation state of IκBα (Serine p32/36) was analyzed again upon TNF or TRAIL treatment. In this context, by adding our control, TNF or TRAIL a clear increase, but only a weak or absent phosphorylation of IκBα, in case of HOIP (Fig. 3C), HOIL-1 (Fig. 3D), or SHARPIN (Fig. 3E) downregulation was shown. These results clearly indicate that in HEK293 cells, all the LUBAC components together are crucial for basal and TRAIL- as well as TNF-stimulated NF-κB induction, but HOIP seems to play a most important role (similar as it was shown with TNF).

## Discussion

In this study, we were able to show that ABIN-1 as well as ABIN-1-MAD inhibit TRAIL-based NF-κB signaling. This finding is supported by experiments in HEK293 and HeLa (unpublished data) cells, in which downregulation of ABIN-1 by miRNA leads to significant higher TRAIL-based NF-κB transcriptional activity. The ability of ABIN-1 to inhibit this pathway after induction with TNF has already been shown previously.^25^ ABIN-1 usually interacts with the deubiquitinase A20 and binds to polyubiquitylated NEMO (IKKγ).

The exact role of A20 as key inhibitor of TRAIL-induced NF-κB signaling working as deubiquitinase or as a binder of linear ubiquitylation is still under debate.^22^ Considering the potential results of this study that ABIN-1 may inhibit NF-κB signaling even without A20 could mean that ABIN-1 may work as binder of the linear ubiquitylation chains and A20 primarily contributes as deubiquitinase for K63-linked chains to achieve NF-κB signaling downregulation. But the exact function of ABIN-1 has to be evaluated in more substantial experiments.

In addition, in this study, we assessed the influence of the LUBAC components HOIL-1, HOIP, and SHARPIN on NF-κB induction levels. In overexpression experiments, we showed that LUBAC is able to potentiate NF-κB signaling upon TRAIL application in dual luciferase and analyzing the IκBα phosphorylation status in western blotting assays. Finally, in dual luciferase assays as well as in western blotting assays, we demonstrated that LUBAC components are required to activate this pathway by using specific miRNAs against each component, resulting in clear NF-κB downregulations and reduced phosphorylation of IκBα on protein level. Hence, we conclude that LUBAC is critically involved in the canonical activation of NF-κB signaling.

The involvement of LUBAC on NF-κB induction has been shown in numerous studies supporting the theory that LUBAC seems to be important for canonical NF-κB activity in general not only in context of receptors.^27,29^ But the exact mechanism of NF-κB upregulation in combination with TRAIL leading to p65/p50 activation is not yet entirely clear. It seems to be that upon Caspase-8 activation a NF-κB activation complex is formed consisting of ubiquitylated RIP1, TAK1, TAB1/2, and the kinases IKKα, IKKβ, and IKKγ (NEMO).^7,19,20,29^ Part of this process is the phosphorylation of IKKα and/or IKKβ to achieve the phosphorylation of IκBα (p32/36) and the activation of p65/p50.^19,20,38,39^

Looking at the results of this study, SHARPIN, HOIL-1, and HOIP also seem to be involved in the activation of NF-κB upstream of the phosphorylation of IκBα in HEK293 cells as shown in other cell lines.^29^ Possibly LUBAC acts in a similar way as it is doing upon TNF signaling by stabilizing the IKK/TAK1 complex to reach efficient NF-κB induction. However, more experiments need to be performed to demonstrate the involvement of linear ubiquitylation and a possible stabilization of this complex. Also, its assembly and its exact position working not downstream or upstream need to be examined.

However, for future applications especially for possible cancer treatments, the finding that LUBAC as well as ABIN-1 are strongly involved in regulation of TRAIL-based NF-κB signaling in HEK293 cells could lead to new therapeutic options. Therapies inhibiting the function of LUBAC, and therefore the TRAIL-induced “canonical” NF-κB activation creating a proapoptotic instead of an antiapoptotic state in cancer cells, is conceivable. Overall, our experiments indicate that ABIN-1 and ABIN-MAD as well as of LUBAC and its components are involved in regulation of TRAIL-induced NF-κB signaling pathway in HEK293 cells.

## Conclusions

Our results show that overexpression of LUBAC components leads to a clear upregulation of TNF as well as TRAIL-induced NF-κB signaling in HEK293 cells. In addition, downregulations of each LUBAC component result in a significant decrease of TNF and TRAIL-induced NF-κB signaling, respectively. In contrary, overexpression of ABIN-1 or ABIN-MAD (not able to bind to deubiquitinase A20) leads to significantly lower and downregulation of ABIN-1 and leads to significantly higher TRAIL-induced NF-κB signaling. All these results clearly indicate that LUBAC is critically involved in initiation and ABIN-1 potentially works as an antagonist of TRAIL-induced NF-κB signaling in HEK293 cells.

Therefore, cancer therapies inhibiting the function of LUBAC and the TRAIL-induced “canonical” NF-κB activation, making them more sensitive for TRAIL treatment, would be a promising approach in case of resistance formation.

## Supplementary Material

Supplemental data

## Abbreviations Used

ABIN-1: A20 binding and inhibitor of NF-kappaB
ABIN-MAD: minimal active domain of ABIN
c-FLIP(L), c-FLIP(s): FLICE-like inhibitory proteins (long, small versions)
cIAP1, 2: cellular inhibitor of apoptosis protein 1
FADD: Fas-associated protein with death domain
HEK293 cells: human embryonic kidney 293 cells
HOIL-1: heme-oxidized IRP2 ubiquitin ligase 1
HOIP: HOIL-1 L-interacting protein
IKKα, IKKβ, IKKγ: IκB kinases α, β, γ
LUBAC: linear ubiquitin chain assembly complex
miRNA: micro RNA
NIK: NF-kappaB-inducing kinase
RIP1: receptor-interacting protein 1
SHARPIN: shank-associated RH domain-interacting protein
TAB1/2: TAK1-binding proteins 1, 2
TAK1: transforming Growth Factor-Beta-Activated Kinase 1
TNF: tumor necrosis factor
TNFR-1: TNF receptor type1
TRADD: tumor necrosis factor receptor type 1-associated death domain protein
TRAF2: TNF receptor-associated factor 2
TRAIL: TNF-related apoptosis-inducing ligand
TRAIL-R1, TRAIL-R2: TRAIL receptors 1, 2
