# Supplementary material for: LUBAC and ABIN-1 Modulate TRAIL-Based NF-κB Induction in Human Embryonic Kidney 293 Cells
Source: Biores Open Access. 2018 May 1;7(1):81–9. doi: 10.1089/biores.2018.0006 (PMC5982153; doi:10.1089/biores.2018.0006)
Supplement: Supplemental data [file Supp_Data.pdf]

Supplementary Data

SHARPIN: miRNA1:top: 5′TGCTGTTCAAGTGTGACCTGCAGCCTGTTTTGGCCACTGACTGACAGGCTGCATCACACTTGAA3′, bottom: 5′CCTGTTCAAGTGTGATGCAGCCTGTCAGTCAGTGGCCAAAACAGGCTGCAGGTCACACTTGAAC3′; miRNA2:top: 5′TGCTGAGGCAGCGTCTTCAAGTGTGAGTTTTGGCCACTGACTGACTCACACTTAGACGCTGCCT3′, bottom: 5′CCTGAGGCAGCGTCTAAGTGTGAGTCAGTCAGTGGCCAAAACTCACACTTGAAGACGCTGCCTC3′; miRNA3:top: 5′TGCTGAGAGGCAGCGTCTTCAAGTGTGTTTTGGCCACTGACTGACACACTTGAACGCTGCCTCT3′, bottom: 5′CCTGAGAGGCAGCGTTCAAGTGTGTCAGTCAGTGGCCAAAACACACTTGAAGACGCTGCCTCTC3′, miRNA4: top:5′TGCTGAGCAAGTAGAGGAAAGCAGGGGTTTTGGCCACTGACTGACCCCTGCTTCTCTACTTGCT3′, bottom: 5′CCTGAGCAAGTAGAGAAGCAGGGGTCAGTCAGTGGCCAAAACCCCTGCTTTCCTCTACTTGCTC3′, miRNA5: top: 5′TGCTGACAGCAAGTAGAGGAAAGCAGGTTTTGGCCACTGACTGACCTGCTTTCCTACTTGCTGT3′, bottom: 5′CCTGACAGCAAGTAGGAAAGCAGGTCAGTCAGTGGCCAAAACCTGCTTTCCTCTACTTGCTGTC3′

HOIP:

miRNA1:top: 5′TGCTGTATTTCTCCAGGATGTTCAGAGTTTTGGCCACTGACTGACTCTGAACACTGGAGAAATA3′, bottom: 5′CCTGTATTTCTCCAGTGTTCAGAGTCAGTCAGTGGCCAAAACTCTGAACATCCTGGAGAAATAC3′; miRNA2:top: 5′TGCTGTAAACTTGACACCACGCCAGTGTTTTGGCCACTGACTGACACTGGCGTTGTCAAGTTTA3′, bottom: 5′CCTGTAAACTTGACAACGCCAGTGTCAGTCAGTGGCCAAAACACTGGCGTGGTGTCAAGTTTAC3′; miRNA3:top: 5′TGCTGATATAATCGCAGCACATCTCGGTTTTGGCCACTGACTGACCGAGATGTTGCGATTATAT3′, bottom: 5′CCTGATATAATCGCAACATCTCGGTCAGTCAGTGGCCAAAACCGAGATGTGCTGCGATTATATC3′; miRNA4:top: 5′TGCTGATCTCGGGAATCCACCACCAAGTTTTGGCCACTGACTGACTTGGTGGTATTCCCGAGAT3′, bottom: 5′CCTGATCTCGGGAATACCACCAAGTCAGTCAGTGGCCAAAACTTGGTGGTGGATTCCCGAGATC3′; miRNA5:top: 5′TGCTGAGAAGTAGCTGAGCAACTGTGGTTTTGGCCACTGACTGACCACAGTTGCAGCTACTTCT3′, bottom: 5′CCTGAGAAGTAGCTGCAACTGTGGTCAGTCAGTGGCCAAAACCACAGTTGCTCAGCTACTTCTC3′

HOIL-1:

miRNA1:top: 5′TGCTGAGCAGATAGAGGTAGGCACTGGTTTTGGCCACTGACTGACCAGTGCCTCTCTATCTGCT3′, bottom: 5′CCTGAGCAGATAGAGAGGCACTGGTCAGTCAGTGGCCAAAACCAGTGCCTACCTCTATCTGCTC3′; miRNA2:top: 5′TGCTGTAGAAATCGCTGGTAATCCTCGTTTTGGCCACTGACTGACGAGGATTAAGCGATTTCTA3′, bottom: 5′CCTGTAGAAATCGCTTAATCCTCGTCAGTCAGTGGCCAAAACGAGGATTACCAGCGATTTCTAC3′; miRNA3:top: 5′TGCTGTGCAATGGTAGCTGAAGGCACGTTTTGGCCACTGACTGACGTGCCTTCCTACCATTGCA3′, bottom: 5′CCTGTGCAATGGTAGGAAGGCACGTCAGTCAGTGGCCAAAACGTGCCTTCAGCTACCATTGCAC3′, miRNA4:top: 5′TGCTGACATCATCCTCAAAGAAGCACGTTTTGGCCACTGACTGACGTGCTTCTGAGGATGATGT3′, bottom: 5′CCTGACATCATCCTCAGAAGCACGTCAGTCAGTGGCCAAAACGTGCTTCTTTGAGGATGATGTC3′; miRNA5:top: 5′TGCTGTTGACGTGGAAACACACAGGGGTTTTGGCCACTGACTGACCCCTGTGTTTCCACGTCAA3′, bottom: 5′CCTGTTGACGTGGAAACACAGGGGTCAGTCAGTGGCCAAAACCCCTGTGTGTTTCCACGTCAAC3′

ABIN-1:

miRNA:top: 5′TGCTGTTCTGACGCAGCTCAGTGATCGTTTTGGCCACTGACTGACGATCACTGCTGCGTCAGAA3′, bottom: 5′CCTGTTCTGACGCAGCAGTGATCGTCAGTCAGTGGCCAAAACGATCACTGAGCTGCGTCAGAAC3′
